# A companion to the preclinical common data elements for phenotyping seizures and epilepsy in rodent models. A report of the TASK3‐WG1C: Phenotyping working group of the ILAE/AES joint translational task force

**DOI:** 10.1002/epi4.12676

**Published:** 2022-12-13

**Authors:** Melissa Barker‐Haliski, Julika Pitsch, Aristea S. Galanopoulou, Rüdiger Köhling

**Affiliations:** ^1^ Department of Pharmacy, School of Pharmacy University of Washington Seattle Washington USA; ^2^ Department of Epileptology University Hospital Bonn Bonn Germany; ^3^ Saul R. Korey Department of Neurology, Isabelle Rapin Division of Child Neurology, Laboratory of Developmental Epilepsy Albert Einstein College of Medicine Bronx New York USA; ^4^ Dominick P Purpura Department of Neuroscience, Isabelle Rapin Division of Child Neurology, Laboratory of Developmental Epilepsy Albert Einstein College of Medicine Bronx New York USA; ^5^ Oscar‐Langendorff‐Institut für Physiologie Universitätsmedizin Rostock Rostock Germany

**Keywords:** electroencephalography, preclinical models, seizure monitoring, seizure semiology

## Abstract

Epilepsy is a heterogeneous disorder characterized by spontaneous seizures and behavioral comorbidities. The underlying mechanisms of seizures and epilepsy across various syndromes lead to diverse clinical presentation and features. Similarly, animal models of epilepsy arise from numerous dissimilar inciting events. Preclinical seizure and epilepsy models can be evoked through many different protocols, leaving the phenotypic reporting subject to diverse interpretations. Serendipity can also play an outsized role in uncovering novel drivers of seizures or epilepsy, with some investigators even stumbling into epilepsy research because of a new genetic cross or unintentional drug effect. The heightened emphasis on rigor and reproducibility in preclinical research, including that which is conducted for epilepsy, underscores the need for standardized phenotyping strategies. To address this goal as part of the TASK3‐WG1C Working Group of the International League Against Epilepsy (ILAE)/American Epilepsy Society (AES) Joint Translational Task Force, we developed a case report form (CRF) to describe the common data elements (CDEs) necessary for the phenotyping of seizure‐like behaviors in rodents. This companion manuscript describes the use of the proposed CDEs and CRF for the visual, behavioral phenotyping of seizure‐like behaviors. These phenotyping CDEs and accompanying CRF can be used in parallel with video‐electroencephalography (EEG) studies or as a first visual screen to determine whether a model manifests seizure‐like behaviors before utilizing more specialized diagnostic tests, like video‐EEG. Systematic logging of seizure‐like behaviors may help identify models that could benefit from more specialized diagnostic tests to determine whether these are epileptic seizures, such as video‐EEG.

**Key Points**

- The ILAE/AES Joint Translational Task Force identified common data elements (CDEs) for phenotyping seizure‐like behaviors in rodents.
- A case report form (CRF) for phenotyping seizure‐like behaviors in rodents is included for use by investigators.
- These CDEs may harmonize reporting of behavioral features and characteristics associated with acute and chronic seizure‐like behaviors.
- The use of phenotyping CRFs and CDEs could identify models that could benefit from more specialized diagnostic tests, such as video‐EEG.

## 1 INTRODUCTION

Epilepsy is a neurological disorder that affects an estimated 65 million people worldwide. Characterized by spontaneous seizures, cognitive deficits, and neuropsychiatric comorbidities, epilepsy is a chronic condition for which no cure presently exists.^1^, ^2^, ^3^ In contrast to other neurological conditions,^4^ however, rodent seizure and epilepsy models have enjoyed a relatively high degree of predictive success in the identification of many therapeutic compounds for the symptomatic management of seizures^5^; eg, over 30 antiseizure medications (ASMs) are currently available to patients.^6^ Indeed, the identification of new ASMs for the symptomatic treatment of epilepsy has, since 1937, largely relied on the demonstration of in vivo antiseizure effects in one or more rodent seizure and epilepsy models.^7^ Additional animal models have been developed since Merritt and Putnam first applied the cat maximal electroshock (MES) seizure test to provide the initial identification of anticonvulsant efficacy of phenytoin in 1937.^8^ Several models have helped to successfully identify clinically useful therapies; eg, the MES and subcutaneous pentylenetetrazol (scPTZ) acute seizure tests, the genetic absence epileptic rat from Strasbourg (GAERS) model of spike‐wave discharges,^9^, ^10^ as well as the kindling model.^5^ ASMs approved by the United States Food and Drug Administration (FDA) were initially found effective in these models and ultimately demonstrated clinical utility for patients with epilepsy. As a result of this success, these four models have provided a high degree of predictive power in identifying new ASMs, while also establishing a basis for phenotyping seizures and epilepsy‐related behaviors in new preclinical models.

Ultimately, the field is constantly searching for new models that closely resemble clinical epilepsy syndromes or conditions that are also characterized by seizures (discussed below). Rigorous and detailed phenotyping of new and existing models is providing critical insight into the mechanistic basis and causes of seizure‐like behaviors, seizures, and epilepsy. Importantly, serendipitous observations of seizure‐like behaviors in new models may prompt the utilization of more specialized diagnostic tests, like video electroencephalography (EEG) to ascertain whether there is an epileptic origin or explore new mechanisms underlying hyperexcitability and epilepsy. Furthermore, an unexpected observation that an investigational compound reduces or increases seizure‐like behaviors may trigger additional investigations for the compound's antiseizure effects or its safety. Nonetheless, well‐characterized animal seizure and epilepsy models have been, and will continue to be, incredibly important to the study of epilepsy, as well as the successful identification of novel therapies for this and other conditions wherein ASMs can be useful.^11^

Lab‐to‐lab variability is a major issue hampering the successful identification and development of novel therapies, and the advanced understanding of the mechanistic basis of neurological diseases,^12^ including epilepsy and seizures. Harmonization in seizure endpoint reporting and analysis would streamline data collection and interpretation by both established epilepsy researchers and investigators new to the field. There are conflicting methodologies and protocols for epilepsy models and seizure reporting, which can be particularly challenging for inexperienced researchers, as well as between different laboratories. For example, the concordance of gene expression changes in status epilepticus (SE) models was only 26‐38% between laboratories and 4.5% between models,^13^ pointing to a degree of variability, which complicates transcriptomic data interpretation and supports the need for transparent and rigorous reporting structures. There are also differences in the reporting of seizure endpoints in acute seizure models. For example, timed seizure duration versus presentation of specific behavioral characteristics is an endpoint in the commonly used 6 Hz model of focal seizures, which has been pharmacologically well‐characterized in mice and rats.^14^, ^15^, ^16^, ^17^, ^18^ Differences in diet can markedly affect the presentation of acute seizures and neuropathology in a model of CNS infection‐induced epilepsy.^19^ Animal strain and sex can substantially alter seizure phenotypes and model fidelity,^20^, ^21^, ^22^, ^23^, ^24^ as well as grossly impact ASM efficacy.^17^, ^20^, ^25^ Lastly, there is a widespread variability in the use of the Racine stage seizure scoring and behavioral seizure phenotyping system across laboratories, which can markedly impact reproducibility, data interpretation, and resulting conclusions. These subtleties can altogether create significant difficulties for new investigators interested in pursuing epilepsy‐related research, as well as hamper the widespread uptake and of new epilepsy models.

The implementation of common data elements (CDEs) for seizure phenotyping in animal models goes beyond epilepsy research alone. Such efforts may, as a result, also improve the study and/or management of numerous other neurological diseases that are associated with abnormal network hyperexcitability. Seizures occur in several other diseases and thus in corresponding rodent models of these diseases. As an example, rodents with Alzheimer's disease‐related genetic variants are well‐known to experience spontaneous seizures,^26^, ^27^, ^28^, ^29^, ^30^, ^31^ which are also observed clinically.^32^ Seizures can occur as a comorbidity to various neurological insults, and it is important to characterize these seizures to better understand the neuropathological implication and pathological mechanisms of the disease. For example, animals subjected to experimental stroke and/or traumatic brain injury can frequently also present with seizures during the acute recovery period,^33^, ^34^ consistent with human clinical presentation.^35^ Gliomas will consistently elicit seizures in human and animal models.^36^, ^37^ Acute viral infection of the central nervous system can provoke seizures in human and preclinical models,^21^, ^38^, ^39^, ^40^, ^41^, ^42^, ^43^, ^44^, ^45^, ^46^, ^47^ providing valuable insight into inflammation‐mediated mechanisms underlying seizures and epilepsy. In addition, intraventricular infusion of patient autoantibodies^48^, ^49^ or T‐cell‐mediated encephalitis^50^ can induce autoimmune‐associated epilepsies in mice. Viral‐induced encephalitis by infection with Theiler's mouse virus (TMEV) can cause seizures and epilepsy.^38^, ^51^ Toxicity induced by acute organophosphate (OP) exposure is often used to elicit a potentially fatal cholinergic crisis associated with severe seizures, which rapidly progress to status epilepticus (SE) in preclinical models.^52^, ^53^, ^54^, ^55^ These OP models are critical for identifying medical countermeasures to chemical threats that pose significant worldwide risks to human health.^52^, ^53^ Ultimately, seizures represent a defining feature not only in epilepsy but also in numerous other clinical disorders, making a unified approach to phenotyping spontaneous or evoked seizure events in preclinical models of paramount importance for the effective and efficient study of many neurological diseases.

**FIGURE 1:**
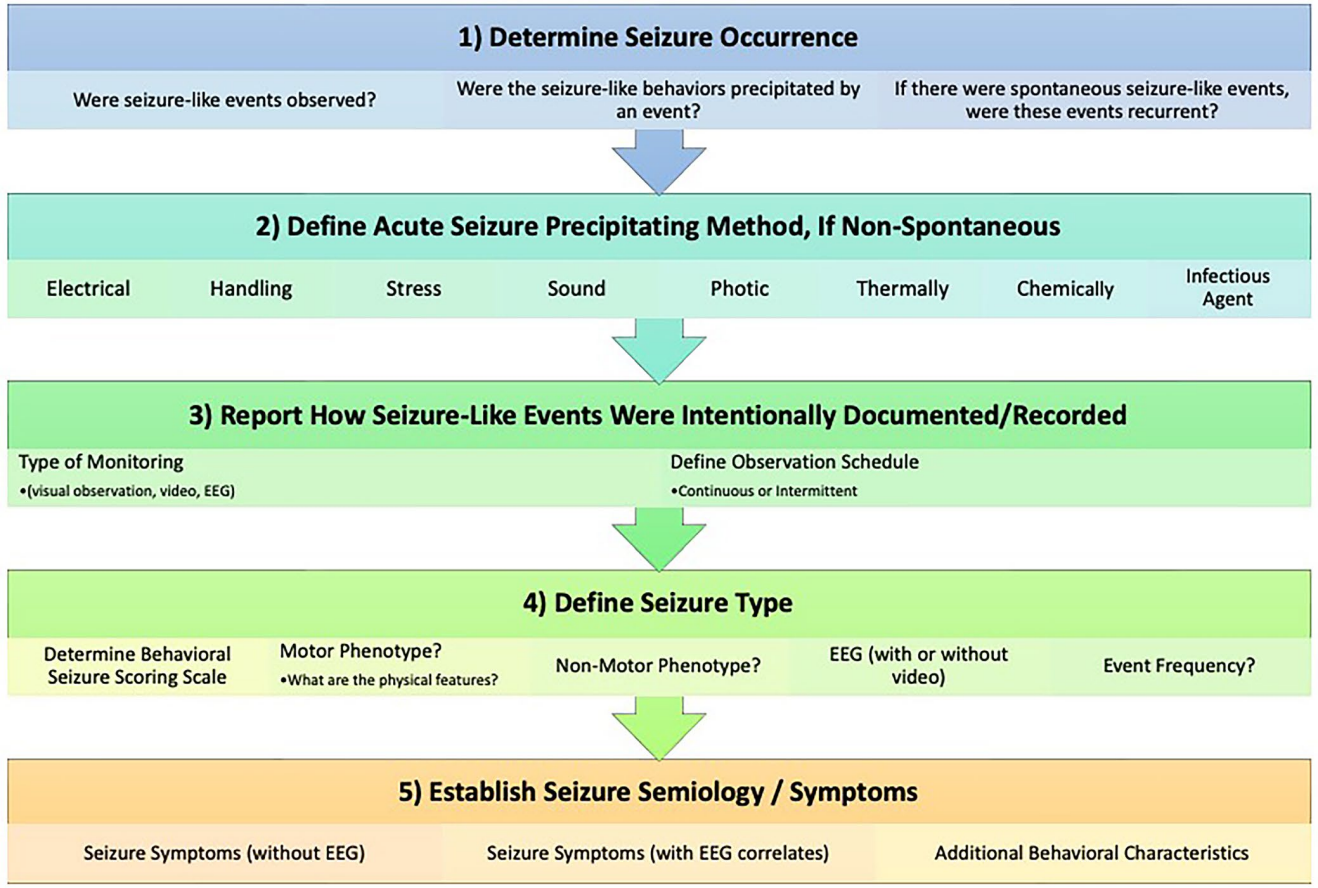
Proposed evaluation and documentation procedure for any novel animal model for which hyperexcitability, seizures, or epilepsy is suspected to arise as a result of genetic, manipulation, behavioral, or chemical means. Importantly, this approach emphasizes the use of EEG in the late characterization of seizure phenotyping in new animal models for which suspicion of seizures exists. The reader is referred to the companion case report form to determine core data elements that should be collected at each of these detailed steps to phenotype a novel rodent model.

Seizures can also be confused with other behavioral abnormalities in laboratory rodents. Although visual screening for seizure‐like behaviors may not suffice to diagnose epileptic seizures, it may select preclinical models that would benefit from video‐EEG. It may subsequently allow comparisons across models, which could inform on model‐specific behaviors that could be further explored mechanistically. In this regard, a good classification and phenotyping system can increase reproducibility between laboratories and within and between investigative teams. The ILAE/AES sought to generate a follow‐up series of CDEs and CRFs that could complement documents already generated for epilepsy researchers so as to improve the strength and reproducibility of epilepsy studies across laboratories.^56^, ^57^, ^58^ The goal of the TASK3‐Working Group 1C (WG1C) was to thus identify and develop CDEs and an accompanying case report form (CRF) to provide investigators of all experience and resource levels with a template that may facilitate the harmonization of essential information on the phenotyping of seizure‐like behaviors in new and existing animal models across laboratories. Considering that some investigators may observe seizure‐like events in an animal model as a result of experimental manipulations without any preceding experience in performing video‐EEG recording, and may or may not possess the resources and/or expertise to conduct such recordings, the accompanying CRF is meant to offer a guidance document for seizure phenotyping with or without EEG monitoring. Because it may not initially be known whether an event constitutes an actual seizure or epilepsy‐related phenotype in a new model, or merely results from experimental manipulation, strictly speaking, one should refer to potential events as “seizure‐like.” For the sake of simplicity, however, in this manuscript, we maintain the term “seizure” for such potential seizure‐like events. However, the readers should bear in mind that any behavior can only be referred to as a “seizure” once it has been definitively classified as such via expert witness, video recordings, or EEG. We herein propose a systematic behavioral and electrographic seizure phenotyping procedure to improve reporting across groups (Figure 1). It is anticipated that this effort will improve the future cost‐effectiveness, scientific strength, and reproducibility of the studies performed wherever new or existing preclinical models are applied that are characterized by seizures or epilepsy‐related behavioral features. This companion document also provides background information on the importance of the various fundamental elements relevant to rigorous behavioral phenotyping.

## 2 METHODS AND PROCESS TO GENERATE CDES AND CRFS

Numerous etiologically relevant and well‐described rodent models of acquired epilepsy, genetic encephalopathies, infection‐related epilepsy, and trauma‐induced epilepsy have provided an important platform for hypothesis testing and target validation to better understand the processes underlying ictogenesis, epileptogenesis, and epilepsy. However, there is a high degree of heterogeneity between researchers and across laboratories in the characterization and application of these models. This variability in reporting and application of the models can lead to incorrect conclusions, which significantly slows therapeutic innovation and advances in clinical care, increases costs, and reduces efficiency in identifying new treatments. In response to these growing concerns,^12^ the ILAE/AES began to develop CDEs for studies of seizures and epilepsy in 2018.^56^, ^58^ Subsequent WGs have since been formed to further expand on this goal. The primary focus of this WG1C was to establish a CRF and identify CDEs for the visual, behavioral, and finally if necessary or feasible, electrographic phenotyping of seizure‐like behaviors in rodent models, regardless of whether it is known to be an actual seizure. While other species (eg, zebrafish^59^) certainly present with behavioral seizures in response to chemical or electrically administered insults, including demonstrated suitability for ASM discovery,^60^, ^61^ similarly establishing such CDEs for nonmammalian systems was beyond the scope of this current WG1C charge. The primary goal was to create a document that could be useful to a nonspecialist (eg, undergraduate, new graduate student, new investigator from outside the epilepsy space, etc.). Effort was made to also minimize the investigator burden. In this present manuscript, we have developed CDEs for seizure phenotyping in rodents. CDEs provide a framework on which to improve the standardization of experimental designs across researchers and laboratories, allowing for different sites to replicate experiments, validate, and ultimately improve the translation of insights gained from rodent models into the clinic.^62^, ^63^

Based on the methodology and approaches previously detailed,^56^, ^57^, ^62^, ^63^ the current TASK3‐WG1C met from 2019 to late 2021 to identify, develop, and refine a core set of CDEs that were considered critical for the successful characterization of incident seizure‐like behaviors in rodents. Through quarterly group teleconferences, the WG1C refined the CRF document to condense it into a usable format. The WG reviewed common study reporting formats and the inclusion of datasets needed to characterize and phenotype a new model. Regardless of the implementation strategy, a core series of CDEs were identified that are relevant to the characterization and phenotyping of seizures, establishing and refining a new or established model with seizures, or characterizing seizure‐associated behavioral manifestations. These CDEs were included in the CRF, and a description of their application is included in this companion document.

## 3 THE CDES FOR SEIZURE PHENOTYPING: OVERVIEW AND UTILIZATION OF THE CRF

CRF File name: CRF_module Phenotyping (Appendix 1)

CDE File name: CDE_chart Phenotyping (Appendix S1).

CRFs can be converted to an electronic format, using the CDEs. Different CRF modules can be combined as needed based on the experimental design. The CRFs previously created by our Task Force allow for linkage between documents and various CRF modules (eg, Core,^58^, ^62^ Behavior,^57^ Pharmacology,^56^ Physiology,^64^ or EEG^65^). If necessary, the electronic forms can be completed by more than one investigator within a laboratory as repeatable modules, whether in the electronic database or in the manually completed CRFs. A summary CRF vetted for accuracy can be created to combine individual observations.

Each component of the CRF is assigned a priority level—High (H), Intermediate (I), or Low (L)—to guide the researcher on the relative importance of recording each data point. For example, documenting the prior surgical history of an animal is of high importance if seizures are reported postsurgery; seizures can be acutely associated with intracerebral manipulation, including EEG electrode implantation, infusion of investigational agents, or therapeutic agents as a treatment‐emergent adverse event of the agent itself.

### 3.1 Seizure occurrence

This section (section I of CRF) is critical for describing the observed seizure‐like event. This section would be used in the initial reporting of seizure‐like events after some experimental manipulation and would likely be the first behavioral features to be systematically assessed by a research group. The investigator can indicate in this section whether the observed event was evoked, and if so, how and in what time frame the seizure‐like event then occurred in relation to a precipitating event. The information in this section of the CRF is essential to data interpretations and should always be completed in full. Much of this section is considered “High” importance for reporting and documentation purposes. Seizures may be precipitated or spontaneous, with the latter also including single/isolated or recurrent events. When it is not known whether these seizure‐like events have an attributable trigger, the investigator should simply indicate “Unknown” until further details can be clearly obtained (ie, there is insufficient temporal or contextual evidence to clearly ascribe a causative precipitating event to the observed seizure). Considering that the CRFs are repeatable modules, an investigator could repeatedly complete this form as additional animals are noted to have seizure‐like events. Events to be defined with the CRF in Section I—Seizure occurrence are:

1. *Were seizure‐like events observed?* First, the investigator should take care to report whether seizure‐like events occurred in the first place. This question is simply meant to classify whether an event was definitively visually observed (“Yes”) or suspected (“Unknown”). The events can be either isolated or potentially more frequent. Nonetheless, the mere observation of seizure‐like events or suspicion of such events should trigger further investigation and detail in the ensuing CRF fields.
2. *If yes, were the seizure‐like behaviors precipitated by an event*? Here, the investigator should indicate whether the observed seizure‐like events were precipitated by a causative trigger, spontaneous, or unknown. This distinction is critical to better determine whether the phenotype represents epilepsy, per se, or a provoked seizure that arises from some precipitating event. In this field, the investigator should provide clues as to whether the observed seizure‐like event is due to previous *seizure‐precipitating* events in the animal's history. These could include daily laboratory routine, such as handling, and treatments such as anesthesia, surgery, administration of medications, or use of a special diet or food. Furthermore, the genetic background^66^, ^67^, ^68^, ^69^ or genetic manipulation (Adeno‐associated virus approaches,^70^ CrispR‐technology,^71^ in utero electroporation,^72^, ^73^ etc) that could trigger seizures should also be indicated as potential causative triggers here. The precise mechanisms leading to an evoked seizure‐like event will require further mechanistic investigation, but the investigator is herein encouraged to document whether any known manipulations could have potentially led to the seizure‐like event. Spontaneous/unprovoked seizures occur in the absence of any provoking cause, implying the absence of a known transient or reversible factor that lowers the seizure threshold and triggers a seizure at that time.^74^, ^75^, ^76^ Epilepsy is characterized by the presence of recurrent spontaneous seizures such that acute, isolated seizures do not necessarily represent an epilepsy phenotype.^75^ The dates and times of any seizure‐like event should be recorded, if known.
3. *If there were precipitating events, when did seizure‐like event(s) occur?* Acutely provoked seizures may occur prior to, acutely, within 24 hours to 7 days of the causative trigger (ie, those that are observed following an electrical, chemical, thermal), or physical stimulus, such as an initiating event (eg, status epilepticus, stroke, or traumatic brain injury), or more than 7 days after an event. The option “immediate, at time of precipitating event” is meant to indicate an acute seizure that is evoked at the time of the triggering event. This applies to different acute seizure models such as PTZ, bicuculline, or maximal electroshock (MES).^77^, ^78^ By contrast, seizures may occur within the first 7 days after the initiating event (eg, induced status epilepticus or traumatic brain injury). In certain models, such as traumatic brain injury or stroke, seizures that occur during the first week after induction are called “early seizures.” In these models, only “late seizures,” occurring after the 7th postinduction day, are considered as spontaneous seizures that can be used for epilepsy diagnosis.^79^, ^80^, ^81^ The investigator should therefore define in this field the temporal relationship between the precipitating event (if any known) and the seizure‐like event. If there are multiple precipitating events, then the investigator should adapt this document according to their needs.
4. *If there were precipitating events, please indicate the type?* Here, the investigator should indicate the type of experimental manipulation that precipitated a seizure‐like event, as best as possible. This can include anesthesia, surgery, drug or investigational agent administration, infectious agent, physical trauma, genetic background/manipulation, diet, handling, a specific seizure induction protocol, or other factor. The date and time of the precipitating event should also be documented in case age is a critical consideration to the seizure‐like event occurence.^82^, ^83^ Importantly, this section should allow for linkage to the CRF modules previously created by our Task Force (ie, Pharmacology^56^) and Pediatrics.^84^, ^85^
5. *If there were spontaneous seizure‐like events, were these events recurrent?* In this field, the investigator is asked to report whether spontaneous events (ie, those without any known precipitating trigger) were recurrent unprovoked events that occurred on separate days, occurred over a single day, or unknown (Table 1).

**TABLE 1:** Terminology used to complete the phenotyping CRF to classify seizure occurrence, seizure induction method, and seizure presentation

Terms	Definition/characteristics	Correlate	References
Epileptic seizure	An epileptic seizure is a transient occurrence of signs and/or symptoms due to abnormal/pathological excessive or synchronous neuronal activity in the brain. These events could be considered recurrent spontaneous/unprovoked seizures.	Definition in human	86
Genetic epileptic seizures	Seizures are believed to be due to known epilepsy‐related genetic variants, as they are observed more frequently in mutant than in wild‐type littermates. These events could be considered recurrent spontaneous/unprovoked seizures.	Genetic epilepsy models	Please refer to TASK3‐WG1B Genetic Models CRF (Mantegazza et al, 2022)^87^
Spontaneous seizures. Absence of known induction, ie, those that do not require an electrical, chemical, thermal, physical stimulus, or genetic manipulation	“Unprovoked” implies the absence of a known transient or reversible factor lowering the threshold and producing a seizure at that point in time. Seizures that occur at distant timepoints after a seizure‐precipitating/induction event (ie, greater than 7 days post‐TBI or poststroke) can also be considered as unprovoked. Model‐specific differences in latency to spontaneous seizures may occur.	Human studies. Induced models	74, 75
Provoked seizures (in humans)	Acute symptomatic seizures also known as reactive seizures, induced seizures, and situation‐related seizures, including those that occur in acute illness. These events could be considered acute provoked seizures.	Human studies	74, 88
Induced seizures (in models)	Induced seizures, ie, those that are observed early after a precipitating event, such as an electrical, chemical, thermal, or physical stimulus but do not indicate epilepsy. These events can be entirely dependent on the seizure model.	eg, PTZ model, maximal electroshock model	5, 89
Early/acute (postinduction) seizures	Seizures that occur in induced models but are insufficiently distant from the inciting event to be considered spontaneous seizures or epilepsy. These events are observed early after an electrical, chemical, thermal, or physical stimulus, up to 7 days after stimulus. These events are considered provoked seizures. Post‐traumatic brain injury and poststroke models can present with seizures due to the insult up to 7 days later; such “early” postinsult seizures are not considered as spontaneous.	eg, post‐TBI or poststroke models	Please refer to TASK3‐WG1B Pediatric Acquired Epilepsies CRF (Katsarou et al, 2022)^77^, ^79^, ^80^, ^81^
Unknown—Unclear/undefined seizures	There is currently insufficient evidence to know what caused the seizure	N/A	N/A

### 3.2 Acute seizure‐precipitating method

This section (section II of CRF) is useful to define whether the seizures that are observed are induced events, in that the investigator can attribute a specific precipitating mechanism to the presentation of seizures. The relevant aspects of this section of the CRF should be completed for an acutely induced seizure. Importantly, the age of animals, whether in a thermally, electrically, or chemically evoked seizure threshold model can introduce substantial variability to the seizure presentation and/or seizure susceptibility, as seizure threshold generally increases with age.^82^, ^90^, ^91^, ^92^ The age of animals used in this procedure, however, should be documented in the General Core CRF wherein the specific animal characteristics (strain, sex, age, etc) are recorded.^58^ For each section below, the relevant information that is needed to evoke a seizure should be reported. The investigator is first encouraged to include the filename of the uploaded seizure‐precipitating protocol file, such that specific methodological details can be documented (eg, stimulation frequency or chemoconvulsant dose, etc.). Seizures can be acutely evoked through numerous methods:

1. *Induction by electrical stimulation*. Electrically evoked seizures are commonly used to induce acute seizures (both mild, generalized, or status epilepticus), or to elicit chronic seizures that model the development or progression of epilepsy. Examples include the validated MES model of acute seizures or the electrically‐induced kindling models of chronic seizures.^5^, ^89^, ^93^, ^94^ Additionally, long‐lasting electrically‐induced seizures can be used to evoke status epilepticus.^95^ Electrically evoked seizures are often the result of intentional induction of seizures in an experimentally‐manipulated animal model (ie, due to genetic variants,^92^, ^96^, ^97^, ^98^, ^99^ or prior neurological insult^40^). Irrespective of the electrical stimulation protocol, the method to elicit the seizure requires the delivery of the electrical stimulus through either auricular, corneal, or implanted electrodes into the brain region of interest. The date and time of induction should also be documented. Therefore, if an electrically evoked seizure is elicited, the investigator should record as such and append relevant Pharmacology^56^ or Pediatric Acquired Epilepsies CRF (Katsarou et al, 2022) files as appropriate. The date and time of induction should be documented on the CRF.
2. *Induction by handling*. Handling‐induced seizures can be elicited in various models that result from genetic or behavioral manipulations.^39^, ^40^, ^51^, ^100^ The date and time of induction should be documented on the CRF.
3. *Induction by stress*. Stress, whether acutely or chronically delivered to preclinical models, can lower the seizure threshold and provoke acute seizures.^101^, ^102^ The type of stress (ie, cage changes/cleaning,^103^ routine husbandry manipulation, restraint, social isolation, etc.), duration, and frequency should be documented in the uploaded induction file. The date and time of induction should be documented on the CRF.
4. *Induction by sound*. Sound is a well‐documented trigger for reflex seizures in several mouse strains, including DBA mice.^82^, ^104^, ^105^ The Frings audiogenic seizure‐susceptible mouse model has been in use to characterize novel investigational agents submitted to the long‐standing NINDS‐sponsored Epilepsy Therapy Screening Program for many years,^106^, ^107^ and represents a useful model of sound‐induced reflex seizures that are beneficial to understand the systemic effects of investigational agents or therapeutic manipulations on seizures susceptibility.^108^, ^109^, ^110^, ^111^ This model in particular is interesting to explore how seizures may arise in genetically‐modified mice developed through selective breeding strategies, including de novo development of seizures of unknown genetic contributors.^112^, ^113^, ^114^, ^115^ Notably, audiogenic seizure induction models have been used for hypothesis testing and antiseizure drug discovery because they provide a reasonable demonstration of brain penetrance of investigational agents.^116^ Further, audiogenic seizures have demonstrated the capacity to induce a kindling phenomenon in rats^117^; highlighting an important consideration for the effect of chronic seizures on the escalating seizure severity. The specific parameters used to evoke a seizure in response to sound should be documented in the uploaded induction file and include frequency, pressure level, and duration. The date and time of induction should be documented on the CRF.
5. *Induction by photic stimulation*. Photic stimulation is useful in clinical studies to initially evaluate potential therapeutic agents for photosensitive seizures in people.^118^ It therefore holds translational utility in preclinical models to evoke seizures. In the CRF, the specific parameters used to elicit a photic stimulation‐induced seizure are necessary to report in the uploaded induction file and include frequency, light intensity, and duration of the stimulation. The date and time of induction should be documented on the CRF.
6. *Induction thermally*. Thermally‐induced seizures are a particularly useful method to evaluate novel therapeutic agents in mouse models of Dravet syndrome, a rare developmental epileptic encephalopathy associated with variants in the *Scn1a* gene.^119^ *Scn1a+/−* mice experience seizures in response to increased body temperature,^83^ consistent with the clinical condition in people. As a result, it is technically feasible to apply a thermally‐evoked seizure induction protocol in a seizure‐susceptible model of genetic epilepsy to evaluate investigational compounds or therapeutic interventions,^120^ which is also broadly relevant to other genetic or insult‐associated models where seizures are observed as a result of changes in body temperature. A detailed protocol to determine the threshold for thermally‐evoked seizures has been previously reported by an earlier ILAE/AES TASK3‐WG (Pharmacology CRF of 2018).^56^ In this CRF, the specific parameters used to elicit a thermally‐induced seizure are necessary to report in the uploaded induction file and include starting temperature, temperature steps and rate, and duration of the stimulation. The date and time of induction should be documented on the CRF.
7. *Induction chemically*. Chemically evoked acute seizures are perhaps the most frequently used method to evoke seizures across laboratories and neurological disciplines throughout the world.^5^, ^121^, ^122^, ^123^, ^124^, ^125^, ^126^, ^127^, ^128^ There are numerous chemical agents that can be used to acutely evoke a seizure when administered by various routes to laboratory animals. We have herein included the most widely used chemical agents to evoke acute/brief seizures (pentylenetetrazol, bicuculline, penicillin, flurothyl, picrotoxin) or status epilepticus (kainic acid and pilocarpine), but this should not exclude other chemical compounds that are available and widely used (ie, organophosphates are not listed but would be subject to the same reporting parameters^52^), are increasingly being used for studies of epilepsy (eg, coriaria lactone^129^), or will be identified in the future. However, it should be emphasized that the chemoconvulsant in use must include several critical reporting parameters when being applied to studies of seizures and epilepsy: route of administration, concentration, dose, and formulation information are all critical to the fidelity of the model across laboratories and thus must be included in the uploaded induction file. Lastly, additional information germane to any sound pharmacological study should be included, which has been previously^56^ and more recently documented (Pediatric Acquired Epilepsy CRFs).^84^
8. *Induction by bacterial or viral infection, or viral introduction of genetic modification*. Central nervous system (CNS) infections are one of the main risk factors for epilepsy in resource‐poor settings.^42^, ^130^, ^131^ Neurocysticercosis is the most common helminthic infection of the CNS and frequently leads to acute seizures and epilepsy, particularly in susceptible regions of the globe.^132^ Several viral infection‐induced epilepsy models exist to study immune system‐mediated seizures and epileptogenesis.^38^ For example, CNS infection of C57BL/6J mice with Theiler's murine encephalomyelitis virus (TMEV) is a valid preclinical model of infection‐induced epilepsy that is well‐suited to elucidate mechanisms of epileptogenesis^133^, ^134^, ^135^ and identify novel pharmacotherapies for the treatment of seizures or prevention of epilepsy.^39^, ^100^, ^136^, ^137^, ^138^, ^139^ Additionally, there is increasing interest in the contributions of autoantibodies^48^, ^49^ and T‐cells^50^ to induce autoimmune‐associated epilepsies. Other agents, such as herpes simplex virus also effectively induce acute seizures in rodents.^140^ Therefore, bacterial or viral infection‐induced encephalitis can evoke acute seizures, and potentially epilepsy. The date and time of infection should be documented on the CRF.

### 3.3 Seizure observation

In the next section of the CRF (section III of CRF), the parameters used to document an apparent seizure‐like event, whether precipitated or spontaneous in nature, should be documented. This section includes recording of the type of monitoring performed (ie, visual observation; video, EEG recording, or other) and the observation schedule (ie, continuous or intermittent). If the seizure observation includes recorded digital files, the relevant filename should be documented. The period of monitoring (start and end time) should be included in this CRF. The investigator is referred to the detailed EEG recording methodology and CDEs previously reported.^65^

### 3.4 Seizure type

This section (section IV of CRF) details the common presentations of behavioral and electrographic seizures in animal models of seizure and epilepsy (Table 2). The investigator is urged to include information on the specific rating scale used to report seizures, with the most commonly used being the Racine scale (and modifications thereof).^141^, ^142^ The motor phenotype of the seizure‐like event is then classified based on the physical manifestations observed. The specific types of behavioral motor phenotype associated with seizures can include tonic (stiffness or rigidity of the entire body or specific limbs^143^), clonic (sustained rhythmic jerking), tonic–clonic, spasms, myoclonic, atonic/falling, running/hyperactivity, behavioral arrest/freezing, or other. Alternatively, seizures can have a nonmotor phenotype and can include features such as immobility, autonomic features that may require specialized equipment to reveal, other symptoms (vocalizations, cognitive, or neuropsychiatric symptoms). Certainly, these nonmotor features may also not be detectable or assessed. In any case, the defining features of the seizure that has been recorded by EEG can also be classified in the next fields of this CRF. If there was an EEG seizure correlate, the location of seizure onset should then be reported, whether it is focal, focal to bilateral, generalized, or unknown in origin. Finally, the field of seizure frequency should document the occurrence profile (single occurrence, rare, monthly, weekly, daily, or unknown) and the duration of the seizure, if recorded.

**TABLE 2:** Terminology of seizure‐like symptoms used in the phenotyping CRF sections IV and V. Behavioral symptoms included in this seizure symptoms table may or may not correlate with EEG recordings

Seizure symptoms	Behavioral features	Further References
Tonic, extension	Most severe phase of a generalized tonic–clonic seizure in which forelimbs and hindlimbs flex then extend into a fixed posture of variable duration, depending on seizure induction method.	144, 145
Tonic, flexion	Penultimate phase of a generalized tonic–clonic seizure in which forelimbs and hindlimbs flex prior to extension.	144, 145
Forelimb clonus	Repeated spastic flexing of forelimbs, which can be rhythmic in nature. Forelimb clonus can follow tonic flexion or extension of a generalized seizure or can precede a tonic component of a seizure. Notably, i.v. PTZ results in first a clonic phase and then a tonic phase (ie, clonic–tonic seizures) whereas MES results in tonic–clonic seizures. Can be unilateral or bilateral, with preserved righting reflex.	78
Hindlimb clonus	Repeated spastic flexing of hindlimbs, which can be rhythmic in nature. Hindlimb clonus can follow tonic flexion or extension of a generalized seizure or can precede a tonic component of a seizure. Can be unilateral or bilateral, with preserved righting reflex.	143
Facial clonus/twitching/chewing	Repeated spastic flexing of facial muscles, ears, or jaw, which can be rhythmic in nature.	143
Spasms	Periods of flexion or extension of the head or limbs, or arching of the entire body. EEG correlation is needed to determine if it is an epileptic seizure.	146, 147
Myoclonus	Jerking of the head, limbs, or entire body—can appear as if the animal has been pushed back because of muscular spasms	143
Mixed response—extension/flexion	Repeated spastic flexing and extension of the limbs.	143
Behavioral arrest/freezing	Brief cessation of normal exploratory behavior and/or activity, which can vary in duration but often no longer than 10 sec. Common in absence models but also defines other models of focal or generalized seizures, fear, or anxiety‐related behavior. EEG correlation is needed to determine if it is an epileptic seizure. A supporting video of behavioral arrest/freezing behavior is included as a Video S1.	39, 100
Immobility	Absence of movement or other activity without clear abrupt cessation of prior activity. It may be normal behavioral state or associated with specific EEG patterns, such as spike‐wave discharges, focal or generalized seizures, or due to pharmacologic effect. EEG correlation is needed to determine if it is an epileptic seizure.	148, 149
Staring	Short staring periods have been associated with spike and wave EEG activity in some rat strains, may be observed with focal or generalized seizure activity, or may be random normal behavior. EEG correlation is needed to determine if it is an epileptic seizure.	150, 151
Head nodding	Immobility and other automatisms accompany this behavior in the early phases of seizure progression, including in Racine stage 1 and 2 seizures. Head nodding is not necessarily epileptic. EEG correlation is needed to determine if it is an epileptic seizure.	141, 152
Barrel twist/rotation	A barrel twist/rotation can be a motor seizure originating from the brainstem and cerebellum. It may, however, present without any EEG seizure correlate, as reported after bicuculline injections. Barrel rotations have been described in models of dystonic behaviors. EEG correlation is needed to determine if it is an epileptic seizure.	153, 154, 155
Rearing	Rearing is less common in rats younger than postnatal day (PD) 15 (see swimming behavior). Defines a Racine stage 4 seizure following bilateral forelimb clonus and can on such occasions be diagnostic of epileptic seizure. Rearing without clonus may occur with normal behaviors, ie, grooming.	141
Falling	Defines a Racine stage 5 seizure when it follows bilateral forelimb clonus and rearing. Falling, without other associated seizure behaviors can occur with other nonepileptic conditions, eg, narcolepsy or other abnormal neurological phenotypes.	141
Violent jumping	Severe stage 5 seizures wherein rearing and falling precedes “popcorn‐like” bouncing activity seizures, consistent with the extended Racine scale of Pinel and Rovner. These seizures have been described as stage 7 seizures in kindling models and KA‐ or PTZ‐induced seizures.	142, 156, 157, 158, 159
Wild running	Common in audiogenic and reflex seizure models wherein the animal will ambulate rapidly and randomly throughout the cage in response to stimulus. Can precede the onset of tonic–clonic seizures in some models.	106, 107
Circling	Preferential circling behavior in a small circumference, which is distinct from wild running (above). This circling behavior may preferentially favor one, both, or alternating sides.	
Straub tail	A vertically erect position of the tail that is elicited through lumbosacral spinal cord via activation of the *Sacrococcygeal dorsalis* muscle. Does not define a seizure but is one of the behavioral features that can define a 6 Hz seizure (the others being stunned or fixed posture and forelimb clonus). EEG correlation is needed to determine if it is an epileptic seizure.	16, 18, 160
Cycling/swimming behavior	Clonic movements can present as cycling swimming behaviors, particularly in pediatric animals based on developmental stage. Rearing is uncommon in young rats (ie, less than postnatal day 15). Young animals do not synchronize clonic movements well, thus up to postnatal day 15, clonic seizures may present as unilateral, rhythmic forelimb movements, or uncoordinated bilateral forelimb movements with occasional hindlimb clonus and repetitive whole‐body jerking. Righting reflex is maintained.	161, 162
Excessive grooming/water licking/sniffing	Automatisms such as grooming, sniffing and water licking can precede or follow a seizure, depending on the inciting mechanism. May or may not have an electrographic component.	
Piloerection	Rough fur coat, which is often characteristic of sympathetic nervous system activation. Often characterizes early stages of seizure onset, especially status epilepticus.	163
Respiratory frequency change	Increase or decrease in respiration rate above a baseline rate. Commonly measured by plethysmography, which directly measures changes in airflow during breathing.	164
Wet dog shakes	Presents as rhythmic, fast alternating shaking of the whole body, beginning at the head, and proceeding in a rostral to caudal manner. This behavior can be elicited as a reflex in response to air being blown on the ear. Ventral dentate granule cell‐dependent mechanism has been proposed in the kainic acid model of status epilepticus. EEG correlation is needed to determine if it is an epileptic seizure.	165, 166, 167, 168
Scratching	Rhythmic and reflexive hindlimb movements elicited without a visible irritating stimulus, which is more common in pediatric models relative to wet dog shakes (ie, within the first 2 weeks in postnatal rats). EEG correlation is needed to determine if it is an epileptic seizure.	161
Heart rate change (If physiological monitoring was done, did heart rate change during a seizure?) If yes, please refer to Physiology CRF	Increase or decrease in heart rate above a baseline rate. Commonly measured by electrocardiogram (ECG), which uses the R–R intervals to analyze heart rate variability, and to assess the conduction of the heartbeat from the sinoatrial node to the ventricles.	169

### 3.5 Seizure semiology/symptoms

Just as in human patients with diverse epilepsy syndromes, animal models of epilepsy can experience either focal onset, generalized seizures, spike‐wave discharges, or a combination. This section (section V of the CRF) should focus on describing the type of seizure presentation that is being reported. This section of the CRF also includes several behavioral features that can be associated with seizures or epilepsy in many animal models (Table 2). This list should not be perceived as an exhaustive list of features associated with every type of seizure across all animal models, but it is provided to offer an investigator (trained or novice) with a core set of CDEs that should be reported whenever a new model is being defined. Table 2 includes a specific description of the individual features associated with the many different types of seizure symptoms. The investigator is encouraged to carefully monitor for the presence or absence of these features and record pertinent and observed symptoms on the CRF.

## 4 SUMMARY AND DISCUSSION

The precise description of the phenotype of a seizure‐like event, whether detected visually, behaviorally, or even electrographically, for the generation of new rod models of seizure and epilepsy is incredibly beneficial to the advancement of our understanding of the processes of epileptogenesis and ictogenesis. However, the precise features associated with any new model wherein seizures may occur, or in a new model of epilepsy, and even for established experimental models, are not always rigorously reported in the initial characterization of the model. In this regard, the adoption and implementation by the field may be misleading, delayed, hampered, or unnecessarily redundant. In this companion manuscript, we have described a series of CDEs and developed a useful CRF to address this knowledge gap. Together, these forms can complement and extend existing CRFs already available in both hard‐copy and electronic format (EPICDE@LONI; https://www.ilae.org/about‐ilae/topical‐commissions/yes/commission‐on‐neurobiology/resources), providing an opportunity to readily integrate data collection, management, and storage in a structured and logical manner. The core goals of this Working Group 1C were to identify the salient behavioral features of a new or established model of epilepsy and/or seizures that should be documented and reported to increase reproducibility and data interpretation. If the investigative team is capable and/or the need arises, this CDE can provide the framework to expand to further electrographic seizure phenotyping with more details provided in the EEG CRF file and companion document.^65^ These CDEs and accompanying CRF are not intended to guide, for example, the ex vivo analysis of pathological features associated with a model (eg, neuroanatomical changes associated with a new model of cortical malformation‐associated mutations^170^) or the pharmacological characterization of a new epilepsy model.^39^, ^171^ In these circumstances, an investigator must append the relevant CRFs (ie, Core,^62^ Physiology,^64^ EEG,^65^ Behavior,^57^ or Pharmacology^56^) as appropriate for downstream endpoints or study objectives. The intention of this present companion document and CRF is that with more rigorous phenotyping of behavioral features of a seizure‐like event in a preclinical model, the degree of reproducibility will be enhanced across diverse researchers and laboratories and allow for improvements in preclinical epilepsy research that will benefit people with epilepsy for decades to come.

## CONFLICT OF INTEREST

AS Galanopoulou who is Editor‐in‐Chief of *Epilepsia Open* and associate editor of *Neurobiology of Disease* has received royalties for publications from Elsevier, Medlink, and Morgan and Claypool publishers. None of the other authors has any conflict to disclose. We confirm that we have read the Journal's position on issues involved in ethical publication and affirm that this report is consistent with those guidelines.

## Supporting information


Appendix S1

Video S1
